# Social Engagement and Cognitive Function of Older Adults in Mexico and the United States

**DOI:** 10.1093/geroni/igaa057.1846

**Published:** 2020-12-16

**Authors:** Bret Howrey, Jaqueline Avila, Brian Downer, Rebeca Wong

**Affiliations:** The University of Texas Medical Branch at Galveston, Galveston, Texas, United States

## Abstract

Social engagement is linked to better cognition, but it is unclear if the social engagement of husbands and wives influences their own cognition as well as each other’s cognition in two very different country contexts. Data on married couples come from the 2001 Mexican Health and Aging Study (MHAS) and the 2000 Health and Retirement Study (HRS), with follow-up cognition measured in 2012. Structural equation models (SEM) were used to test the actor-partner interdependence model on the association of social engagement with cognition. In Mexico wives’ social engagement benefited their own cognition as well as their husbands’, but husband’s social engagement was unrelated to cognition. In the U.S. both wives’ and husbands’ social engagement benefited their own cognition, but not each other’s. Results suggest asymmetric patterns of actor-partner interdependence in Mexico, possibly reflecting more traditional social roles of women and co-dependence within couples, but more independence within U.S. couples.

